# The Effects of L-Arginine in Hypertensive Patients: A Literature Review

**DOI:** 10.7759/cureus.20485

**Published:** 2021-12-17

**Authors:** Abdulkarim W Abukhodair, Walid Abukhudair, Mohammed S Alqarni

**Affiliations:** 1 Medicine, King Saud Bin Abdulaziz University for Health Sciences College of Medicine, Jeddah, SAU; 2 Cardiac Surgery, King Fahd Armed Forces Hospital, Jeddah, SAU; 3 Medicine, King Abdullah International Medical Research Center, Jeddah, SAU

**Keywords:** vasodilation, nitric oxide, endothelium, hypertension, l-arginine

## Abstract

Hypertension (HTN) is a chronic disease that affects more than 972 million people throughout the world, which is usually associated with endothelial dysfunction. Scientists are closely investigating endothelial dysfunction and have recently discovered the endothelium-derived relaxing factor (EDRF) known as NO (nitric oxide), which is derived from a semi-essential amino acid, L-arginine, by the action of endothelial nitric oxide synthase (eNOS). Production of adequate amounts of NO by vascular endothelial cells is essential to maintain normal blood pressure and prevent the development of HTN. Asymmetrical dimethylarginine (ADMA) is an endogenous NOS inhibitor that is increased in those with HTN especially in patients with renal dysfunction. In the present review, the role of L-arginine, arginine transporters, and ADMA in the pathobiology of HTN and their potential clinical significance are discussed.

## Introduction and background

In 2000, a total of 972 million adults were affected with hypertension [1]. Hypertension, the elevation of blood pressure (>140 mmHg systolic and >90 mmHg diastolic), is a worldwide health condition that is responsible for many cardiovascular infirmities such as coronary artery disease, strokes, chronic kidney disease (CKD), and heart failure [2]. It has been named as the leading risk factor for mortality by the Risk Assessment Collaborating Group [3]. In the search of finding an urgent treatment for this chronic disease, researchers discovered that the vascular endothelium is a vital factor affecting blood pressure. Vascular endothelial cells secrete and produce many substances. One of these important substances is nitric oxide (NO), a gaseous compound that consists of one nitrogen molecule and one oxygen molecule, that effectively regulates vascular function. NO is deployed into underlying smooth muscles by the endothelial cells and is considered a vasodilator. Investigating the vasodilator further regarding techniques of augmentation and inhibition prevention is essential in overcoming hypertension. The purpose of this article is to understand the effect of L-arginine on hypertensive patients. We conducted a narrative review of the literature about L-arginine with a special focus on articles that discuss its physiology, its effect on the kidney, and its administrating routes. We discuss the synthesis of L-arginine and its transporters, which affect the physiology of endothelial vasodilation.

## Review

Hypertension and endothelial dysfunction

Hypertension can be caused by the inactivation of NOS and salts, which gradually increases and stimulates blood pressure. Coleman and Guyton conducted an experiment that shows the effect of salts on hypertension in dogs by salt loading, and they concluded that dogs who were salt-loaded acquired an elevation in blood pressure of the arteries, unlike normal dogs [4].

The endothelium, the inner coating of blood vessels, moderates the tone of the vascular smooth muscle by releasing relaxing and contracting factors [5]. NO and endothelium-derived hyperpolarizing factor (EDHF) are two relaxing factors, whereas contracting factors include endoperoxides, thromboxane A2, superoxide anions, and endothelin [5]. It was demonstrated by Nolan Gokce that NO is an essential modulator for the smooth muscles, and it was proven that the absence of the regulator or its synthetic enzyme, NOS, causes an elevation in arterial blood pressure [2]. In many cases, it has been shown that a lack of production of the vasodilator NO by the endothelium leads to a phenomenon known as endothelial dysfunction, which in turn causes hypertension [2]. However, the direct causes of endothelial dysfunction are still unknown and unclear [2].

L-arginine and vasodilation

In 1998, Robert Furchgott, Louis Ignarro, and Ferid Murad were honored with the Nobel Prize in Physiology of Medicine as they discovered the identity of endothelium-derived relaxing factor (EDRF) as NO. It was an important discovery that led to a long series of papers and clinical trials relating to the endothelium vasodilator [6]. NO is produced in vascular endothelial cells from a substrate - the amino acid L-arginine [6]. In the past year, many studies have been conducted to investigate if L-arginine supplementation increases vasodilation and decreases blood pressure. In a study on 29 healthy individuals who were given L-arginine for one week, systolic blood pressure was reduced in 62% of participants compared to baseline hypertensive patients with a mean decrease of 4 mmHg. Also, diastolic blood pressure was reduced in 69% of participants with a mean decrease of 3.7 mmHg. In the 10 participants who were borderline hypertensive (systolic > 130 mmHg or diastolic > 85 mmHg), the mean systolic decrease was 11 mmHg. However, in the 19 normotensive participants, the mean systolic decrease was only 0.22 mmHg. The mean diastolic blood pressure decrease was 4.9 mmHg in borderline hypertensives and 4.5 mmHg in normotensives [7]. Another trial was done on individuals with pulmonary hypertension in which they received a 30-minute infusion of L-arginine. The L-arginine infusion produced a radical decrease in the pulmonary arterial pressure (PAP) by 15.8% ± 3.6% and pulmonary vascular resistance (PVR). In this trial, there was no significant relationship between the baseline intensity of pulmonary hypertension and the amount of the pulmonary vasodilator response to L-arginine [8].

L-arginine is a semi-essential amino acid as it is present in many dietary proteins and produced from another amino acid, L-citrulline. Therefore, an increase in L-citrulline leads to an increase in L-arginine, which leads to an enhancement in NO production. A recent study was done on 17 young men with normal blood pressure who were given citrulline supplementation for four weeks [6]. The result shows that oral citrulline decreased the brachial systolic blood pressure (-6 ± 11 mmHg), aortic systolic blood pressure (-4 ± 10 mmHg), and aortic pulse pressure (-3 ± 6 mmHg) [6]. This study supports that L-citrulline is effectively converted to L-arginine and is an important factor that can increase and improve NO production.

Cellular L-arginine transporters

Extracellular L-arginine needs special transport channels in the plasma membrane to get in the cell. There are two main transport systems, y+ and y+L, which are responsible for transporting L-arginine and cationic and neutral amino acids through the plasma membrane. Cellular L-arginine transport can be affected by extracellular concentration of L-arginine, cationic or neutral amino acids [9]. Both y+ and y+l show competitive inhibition and trans-activation. L-arginine transport can be affected by the extracellular concentration of cationic and neutral amino acids [9]. This happens because other amino acids can compete with L-arginine for cellular uptake [9]. In trans-activation, L-arginine effluence from the cells increases by the addition of cationic or neutral amino acids to the extracellular space [9].

Although the y+ transport mechanism is considered the most vital in epithelial cells, both y+ and y+L transport mechanisms mediate L-arginine uptake. On the other hand, both cationic and neutral amino acids are transported by system y+L. System y+ is a family of cationic transporters that are encoded as CAT1, CAT2A, CAT2B, and CAT3.29-32. The y+l mechanism, which is present in endothelial cells, is connected with CD/984F2 heavy chain. Cationic amino acids of sodium can be independently transported by y+ and y+l systems. However, uptake of neutral amino acids is related to y+l transporters, which depend on sodium [9].

L-arginine transport in endothelial cells is seen to be affected by the concentrations of insulin and glucose. In a research by Sobrevia et al., results showed an augmentation in L-arginine transport due to an elevation in the glucose levels. Insulin was also noted as an important modulator of L-arginine transport. Human insulin stimulated the L-arginine transport in the cells with lower glucose levels while decreasing the transport of L-arginine for the cultured cells with higher levels of glucose. Although K_m_ in the L-arginine transport seems to be greater than that of the latter, this increase was noted as insignificant [10].

Inhibition of nitric oxide synthesis

ADMA is L-arginine’s methylated isomer derived from proteins. ADMA is a competitive inhibitor of NOS. Impaired NO synthesis leads to many conditions [7]. Researches prove that high levels of ADMA are connected with endothelial function inhibition and an increased risk of cardiovascular disease [11]. Individuals with hypertension tend to have poor endothelial function, a condition that can be recovered by the supplementation of L-arginine [12]. Other researches have shown improvement by supplementation of L-arginine in patients with diabetes mellitus and congestive heart failure [13,14]. In a few studies, L-arginine has been shown to benefit men with erectile dysfunction [15].

In healthy middle-aged humans, L-arginine has proved not to be a limiting factor; therefore, it does not correlate with blood significantly [16]. In patients with a deficiency in L-arginine, there was no sign of hypertension. In normal body conditions, the addition of L-arginine does not appear to increase the rate of NO synthesis due to an already saturated NOS [17]. However, the inhibition caused by an increase in the competitive inhibitor of NOS, ADMA, in patients with renal disease, hypercholesterolemia, and arterial disease could be overcome by an increase in L-arginine, dramatically improving the patient’s endothelial dysfunction.

Role of the kidney in hypertension

The main factors of developing renal hypertension are related to the disturbance of the relationship between the renal fluid and renal perfusion pressure and sodium excretion [18]. These renal factors play an essential role in increasing and maintaining hypertension [18]. The development of hypertension is caused due to renal endothelial dysfunction and reduced NO bioavailability [19]. Clearly, there must be harmony between intake and secretion of water and electrolytes. Unless there is a disturbance of renal-pressure natriuresis to higher blood pressures, chronic hypertension will not progress [20].

Most endogenous L-arginine synthesis occurs in the kidney. The kidney acquires approximately 80% of the intestinal citrulline, which is released from glutamine metabolism, for L-arginine production [21]. The bioavailability of NO is increased by L-arginine that increases its formation and reduces its inactivation by superoxides [22,23], due to which L-arginine supplementation is suggested as a potential therapeutic approach in hypertension [9].

L-arginine and angiotensin II-induced hypertension

It has recently been discovered by Rajapakse et al. that an exogenous L-arginine can dramatically reduce angiotensin II (Ang II)-induced hypertension in Sprague Dawley rats [24]. This, we propose, is because Ang II increases the transport of L-arginine and L-lysine and CAT1 mRNA in rat smooth muscles. Because of the increase in CAT1 mRNA and transport of L-arginine, the hypertensive effects of the NO precursor must be increased as well.

While the direct relation of exogenous L-arginine in NO synthesis is still unconfirmed, L-arginine has been discovered to increase renal plasma flow (RPF) in healthy humans by 12%-15% [25]. However, many cases have also been seen to have mixed results depending on whether L-arginine affects Ang II-induced patients. A recent study composed of eight healthy subjects confirmed that L-arginine had almost no effect on their patients [26]. These eight patients received a placebo or a continuous intravenous infusion of Ang II along with a 30-minute administration of intravenous L-arginine. A 10% increase in RPF was noticed in placebo patients; however, the great decrease of RPF by Ang II was not affected by the L-arginine administration.

A recent trial on 10 healthy males studied the effects of L-arginine on angiotensin-converting enzyme (ACE) [27]. An intravenous L-arginine infusion (500 mg/kg over 30 min) reduced serum ACE activity from 10.4 ± 0.6 to 8.9 ± 0.5 nmol/mL.min and plasma Ang II from 19.3 ± 3.3 to 12.7 ± 2.8 pg/mL. In light of this trial, intravenous administration of L-arginine has been observed to inhibit ACE, which in turn decreases the abundance of Ang II. This inhibiting effect might be an explanation for the antihypertensive effects of L-arginine.

Oral administration vs. intravenous administration

Researches have shown us the variation in the results of administrating L-arginine. In Higashi et al.’s trial, 10 healthy males were intravenously (IV) induced with L-arginine (500 mg/kg over 30 min) [27]. As a result, there was a reduction of mean blood pressure from 82 ± 2.5 to 76.3 ± 2.5 mmHg. In an orally administrated L-arginine trial, the mean systemic arterial pressure modestly decreased from 92 ± 4 to 87 ± 3 mmHg. All subjects, whether controlling or hypertensive, tend to have an equal reduction rate in IV administration; although, there is a tendency toward a greater reduction among subjects with higher systolic blood pressure in the oral administrated course. In a trial by Tangphao et al. on 10 healthy patients, L-arginine was intravenously infused for 30 minutes, and L-arginine concentration showed a prompt return to previous baseline levels after the end of the infusion [28]. However, when the same subjects were later administrated orally with L-arginine, L-arginine concentrations did not experience the same drop due to the slow intestinal absorption of L-arginine occurring in the jejunum [28].

## Conclusions

Although the hypotensive effects of L-arginine are not yet confirmed, there is an evident, modest decrease in blood pressure when a patient is administered with L-arginine. A greater decrease in blood pressure is seen in hypertensive patients or patients with endothelial dysfunction. Cellular L-arginine transport can be affected by extracellular concentration of L-arginine, cationic or neutral amino acids. Also, L-arginine transport in endothelial cells is seen to be affected by the concentrations of insulin and glucose. The kidney plays a major role in the synthesis and excretion of L-arginine in terms of reducing blood pressure by acquiring the intestinal citrulline. It is found that L-arginine is better at decreasing blood pressure by reversing the causes of hypertension such as ADMA and Ang II than at improving the blood pressure at regular conditions. L-arginine supplementation has also shown great effects regarding renal hypertension. Individuals with hypertension tend to have a poor endothelial function, a condition that can be recovered by the supplementation of L-arginine. Furthermore, studies show an improvement by supplementation of L-arginine in patients with diabetes mellitus and congestive heart failure. For future references, larger clinical trials must be made to enforce the acknowledgment of the effects of L-arginine. More trials with a specific type of patients would help further understand and explore the effects even more.
